# Novel Usher syndrome pathogenic variants identified in cases with hearing and vision loss

**DOI:** 10.1186/s12881-019-0777-z

**Published:** 2019-05-02

**Authors:** Justin A. Pater, Jane Green, Darren D. O’Rielly, Anne Griffin, Jessica Squires, Taylor Burt, Sara Fernandez, Bridget Fernandez, Jim Houston, Jiayi Zhou, Nicole M. Roslin, Terry-Lynn Young

**Affiliations:** 1 Craig L. Dobbin Research Centre, Discipline of Genetics, Faculty of Medicine, Memorial University, St. John’s, Newfoundland & Labrador AIB 3V6 Canada; 2 Provincial Medical Genetics, Craig L. Dobbin Research Centre, Eastern Health, 300 Prince Phillip Drive, St. John’s, Newfoundland and Labrador A1B 3V6 Canada; 3 Molecular Diagnostic Laboratory, Eastern Health, Craig L. Dobbin Genetics Research Centre, Faculty of Medicine, Memorial University, 300 Prince Phillip Drive, St. John’s, Newfoundland and Labrador A1B 3V6 Canada; 4 The Centre for Applied Genomics, The Hospital for Sick Children, 686 Bay Street, Toronto, Ontario M5G 0A4 Canada

**Keywords:** Syndromic hearing loss, Usher syndrome, RNA splicing, Knowledge translation, Whole exome sequencing, Genetic isolate

## Abstract

**Background:**

Usher syndrome, the most common form of inherited deaf-blindness, is unlike many other forms of syndromic hereditary hearing loss in that the extra aural clinical manifestations are also detrimental to communication. Usher syndrome patients with early onset deafness also experience vision loss due to progressive retinitis pigmentosa that can lead to legal blindness in their third or fourth decade.

**Methods:**

Using a multi-omic approach, we identified three novel pathogenic variants in two Usher syndrome genes (*USH2A* and *ADGRV1*) in cases initially referred for isolated vision or hearing loss.

**Results:**

In a multiplex hearing loss family, two affected sisters, the product of a second cousin union, are homozygous for a novel nonsense pathogenic variant in *ADGRV1* (c.17062C > T, p.Arg5688*), predicted to create a premature stop codon near the N-terminus of *ADGRV1*. Ophthalmological examination of the sisters confirmed typical retinitis pigmentosa and prompted a corrected Usher syndrome diagnosis. In an unrelated clinical case, a child with hearing loss tested positive for two novel *USH2A* splicing variants (c.5777-1G > A, p. Glu1926_Ala1952del and c.10388-2A > G, p.Asp3463Alafs*6) and RNA studies confirmed that both pathogenic variants cause splicing errors. Interestingly, these same *USH2A* variants are also identified in another family with vision loss where subsequent clinical follow-up confirmed pre-existing hearing loss since early childhood, eventually resulting in a reassigned diagnosis of Usher syndrome.

**Conclusion:**

These findings provide empirical evidence to increase Usher syndrome surveillance of at-risk children. Given that novel antisense oligonucleotide therapies have been shown to rescue retinal degeneration caused by *USH2A* splicing pathogenic variants, these solved *USH2A* patients may now be eligible to be enrolled in therapeutic trials.

## Background

Approximately 30% of inherited hearing loss is syndromic and is classically characterized by overt clinical features, such as distinctive craniofacial and eye abnormalities, and joint problems as in Stickler syndrome [1] (MIM: 108300). However, syndromic forms of hearing loss such as Usher syndrome (USH), present more insidiously, often resulting in delayed or misdiagnosis. USH is an autosomal recessive condition characterized by bilateral sensorineural hearing loss with or without vestibular dysfunction, and progressive retinitis pigmentosa (RP) [2–4]. Most children with USH are born with congenital hearing loss; however, progressive RP may present in the second decade, making diagnosis difficult due to the subtle changes in visual function over time [5]. Historically, USH was considered an extremely rare disorder with a frequency of 1 in 25,000 [6]; however, a recent study suggests a higher prevalence of 1 in 6000 individuals in the European (Non-Finnish) population [3].

USH is an extremely deleterious disorder and is the most common cause of inherited deaf-blindness [3]. So far, 13 USH genes have been identified which adversely affect the development of sensory hair cells within the inner ear and of photoreceptors in the eye [5]. The most common subtype, USH type 2A (USH2A), accounts for two-thirds of all cases. Many *USH2A* pathogenic variants cause splicing defects such as exon skipping and the creation or destruction of canonical acceptor and donor splice sites [5]. Novel therapies that target *USH2A* show great promise as retinal degeneration in USH2A patients can be rescued using antisense oligonucleotide-based therapy targeting cryptic splicing variants [7]. Additionally, antioxidant-based therapies have also shown great promise in preventing cone degeneration in USH1 mice, which is linked to oxidative stress [8]. Oxidative stress has well-established roles in many retinal dystrophies, where polymorphisms in *GLO1* may explain RP susceptibility and clinical heterogeneity [9]. Enrollment of patients in therapeutic trials requires a molecular diagnosis which can be challenging in the clinical setting. A comprehensive approach that includes linkage analysis, exome sequencing and functional analysis is often required, especially for novel splicing variants [10, 11]. Herein, we report three novel USH pathogenic variants in *USH2A* or *ADVRG1* identified in cases of vision and hearing loss using a comprehensive multi-omic approach.

## Methods

### Study participants and clinical evaluations

The study involved three families from the Newfoundland population, including two multiplex families. Clinical evaluations included air conduction thresholds using pure-tone audiometry, noting the audiogram configuration, severity, onset and progression. Vision was assessed with ocular examination, visual acuity and visual field testing, electroretinography (ERG) and fluorescein angiography of the retina.

**Fig. 1:**
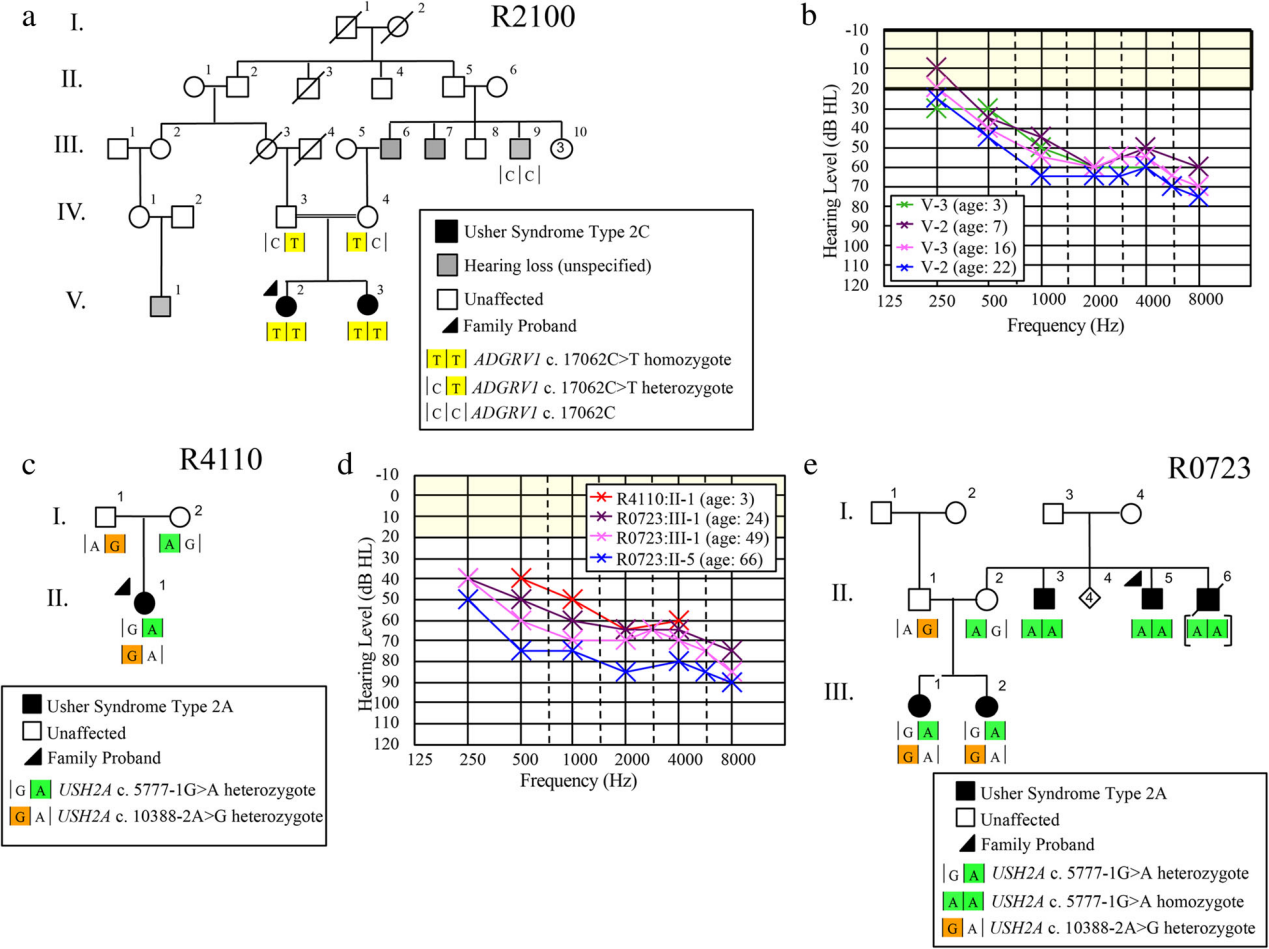
Pedigrees and audiological data of families that were recruited through either a hereditary hearing loss or eye study, or the medical genetics clinic. **a)** Family R2100 is a hereditary hearing loss pedigree with multiple affected sibships. Usher syndrome type 2C was not apparent at ascertainment, **b)** Serial audiograms of PID V-2 and V3 (Family R2100), **c)** Family R4110 was recruited to this study by our local medical genetics clinic. The proband of this family did not pass newborn hearing screens and was *GJB2*-negative, **d)** Audioprofiles of Usher syndrome type 2A families, **e)** Family R0723 is an Usher syndrome pedigree with several affected members

For Family R2100, hearing loss (HL) is present in three sibships with varying audioprofiles, including two sisters who are the product of a consanguineous union (Fig. 1a). The proband (PID V-2) diagnosed with hearing loss at 3 years, presents by age 7 with a mild to moderate bilateral sensorineural HL, and her younger sister (PID V-3) was diagnosed at age 3 with a similar audioprofile (Fig. 1b).

We also recruited a case from our local medical genetics’ clinic (Family R4110) of a child diagnosed at 3 months (following newborn hearing screening), who presents by age 3 with mild to moderate bilateral sensorineural HL (Fig. 1 c, d). In another multiplex family (R0723), the proband and his brothers (PIDs II-5, II-3 and II-6, respectively) were first diagnosed with RP in mid 5th decade when their central vision decreased to the point that they met criteria for legally recognized blindness (Fig. 1e). They reported reduced night vision since the mid-second decade, and hearing loss since young childhood. The proband had been fitted for hearing aids for moderate to severe hearing loss. In R0273, the proband and his brothers (PIDs II-5, II-3 and II-6 respectively) had reported experiencing reduced night vision since their mid-third decade and were all diagnosed with RP in the mid-fifth decade when their central vision decreased. Throughout the course of ongoing clinical assessment, it was noted that the proband was fitted for hearing aids due to a moderate to severe HL and although the age of onset was unknown, he had HL at a young age. Two nieces with early hearing loss were also diagnosed with RP on follow-up, which prompted targeted genetic testing for known USH genes (Fig. 1d).

### Gene panels

In the case of family R0723, a targeted gene panel for 13 USH genes (CEI Molecular Diagnostic Laboratory, Portland, OR, USA) was offered through a research study on hereditary vision loss. In the clinical case of the child who failed newborn hearing screening (Family R4110), the family was offered targeted screening (158 syndromic and non-syndromic hearing loss genes, Blueprint Genetics, Comprehensive Hearing Loss and Deafness Panel, version 1, San Francisco, CA, USA). To validate variants of interest and check for co-segregation with RP and/or HL trait in the families, genomic DNA was amplified using custom primers and sequenced in both directions using standard touchdown PCR protocols (ABI PRISM 3500XL DNA Analyzer; Applied Biosystems, Foster City, CA, USA). Sequence traces were analyzed using Mutation Surveyor Software (version 5.00, SoftGenetics LLC State College, PA 16803).

### Linkage analysis and whole exome sequencing in hearing loss family R2100

We initially screened the proband of Family 2100 for deafness alleles previously identified in the NL population and submitted representative audiograms to Audiogene, a program comparing these to average audiograms of 34 deafness loci [12]. As this targeted approach failed to solve Family 2100, a traditional linkage study was done. For the linkage analysis, we selected three affected and two unaffected relatives (PID V-2, V-3, III-9, and IV-3, IV-4 respectively) (Fig. 1a) and genotyped 17,407 polymorphic markers with the Illumina Human610-Quad chip. Multipoint linkage analysis (Merlin v1.1.2) [13] was performed under a recessive model with a disease allele frequency of 0.07 and a penetrance of 99%. In order to screen candidate genes within linked regions, whole exome sequencing was carried out on 5 family members (two affected offspring and their parents: PID V-2, V-3, IV-3, IV-4 respectively) using the Ion Torrent AmpliSeq RDY Exome Kit (Life Technologies, Cat. #A27193). Purified libraries were loaded onto an Ion Proton PI v3 chip and sequenced with the Ion Torrent Proton. Only rare variants (MAF < 1%) that mapped to linked regions, had a depth of coverage >20X and were of medium to high impact were validated by Sanger sequencing and selected for cascade screening. Population frequencies were determined using 124 ethnically-matched controls.

### Splice variant in silico analysis

For variants of interest that reside within canonical +/− 1 or 2 splice sites, we conducted in silico analyses using Alamut Visual (Interactive Biosoftware Inc., Rouen, France), a program that provides a splicing alteration report by linking to the MaxEnt, NNSPPLICE, SplicSiteFinder, and GeneSplicer algorithms.

### RNA-cDNA validation of splice variants

In order to experimentally validate splicing predictions, we extracted total RNA from B-cell lymphocytes using standard TRIzol-based methods (Thermo-fisher, Cat. #15596026) and prepared cDNA libraries with the Superscript III First Strand Synthesis System (Thermo-fisher, Cat. #18080093). RT-PCR was carried out with primers that spanned candidate splicing regions, followed by TOPO TA-Cloning Kit for Sequencing with One Shot TOP10 Chemically Competent E. coli (Invitrogen, #K457540) according to the manufacturer’s protocol. RT-PCR products were Sanger sequenced and then analyzed using Mutation Surveyor Software (version 5.00, SoftGenetics LLC State College, PA 16803).

## Results

### *ADGRV1* c.17062C > T genotype/phenotype analyses

**Fig. 2:**
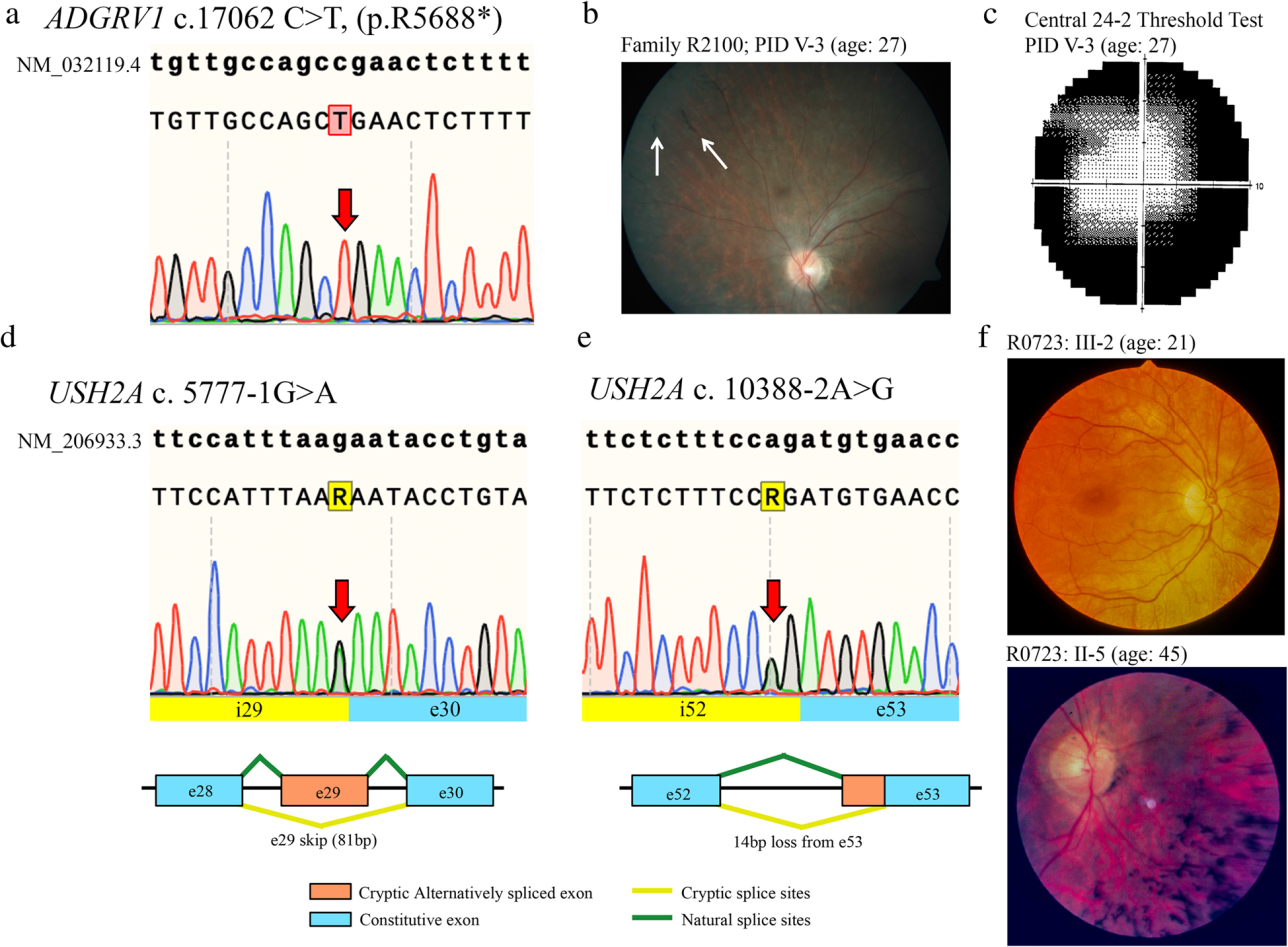
Genomic and clinical findings of families in this study. **a)** Sequence electropherogram of *ADGRV1* c. 17062C > T. Red arrow indicates this homozygous substitution, **b)** Retinal photograph of PID V-3 (Family R2100). White arrows highlight the presence of bone spicules, **c)** Central 24–2 visual threshold test of PID V-3 illustrating a deterioration of peripheral visual acuity, **d)** Sequence electropherogram of USH2A c. 5777-1G > A. This splicing pathogenic variant causes exon skipping and the in-frame deletion of 81 bp, **e)** Sequence electropherogram of *USH2A* c. 10,388-2A > G. This splicing pathogenic variant activates a cryptic acceptor splice site, resulting in the loss of 14 bp and a frameshift (p. Asp3463Alafs*6), **f)** Retinal photograph of PID III-2 at age 21 demonstrates arterial attenuation in the retina, which further deteriorates by the fifth decade as seen in PID II-5 at age 45 (Family R0723)

In the step-wise analysis of hearing loss Family 2100, the proband (PID V-2; Fig. 1a) screened negative for all hearing loss variants previously identified in the NL population. Genome-wide linkage analysis (assuming autosomal recessive inheritance) yielded positive LOD scores suggestive of linkage for 8 genomic regions and the theoretical maximum LOD (1.68) for regions on chromosomes 3, 4, 5, 6 and 15 (Table 1). Subsequently, exome sequencing identified 278 variants that were shared between the proband (PID V-2) and her affected sister (PID V-3). Of these, only eight variants remained after filtering for rare variants (MAF < 1%) of medium to high impact that mapped to linked regions and had a depth of coverage >20X (Table 2). Seven of these variants were shown to be false positive INDELs (did not validate with Sanger sequencing) or did not reside within genes associated with syndromic or non-syndromic HL [14]. The remaining candidate (*ADGRV1* c.17062C > T; p.Arg5688*) is a nonsense variant associated with *USH2C* (Fig. 2a). Co-segregation analysis confirmed that the affected sisters were homozygous for *ADGRV1* c.17062C > T and their parents were unaffected carriers. The only other available affected relative for cascade sequencing was a maternal uncle (PID III-9; Fig. 1a) who was wild-type (two normal copies) and subsequently confirmed to have acquired his hearing loss after a serious diving accident. The nonsense *ADGRV1* variant is predicted to create a premature stop codon nearing the N-terminus of ADGRV1, preventing the translation of all 7 transmembrane domains. Furthermore, the *ADGRV1* c.17062C > T variant is absent in the population controls and has a single heterozygous entry in ExAC browser from the European (Non-Finnish) population. According to ACMG guidelines, the *ADGRV1* nonsense variant should be classified as pathogenic as it meets the following criteria: PVS1, PM2, PM3, PP1, PP3.

**Table 1:** R2100 Multipoint Linkage Analysis Result

		Start		End		
Chromosome	LOD	dbSNP	Position	dbSNP	Position	Size (Mb)
1	0.58	rs3933251	64,095,165	rs591540	71,866,132	7.8
3	1.68	rs1400207	2,825,953	rs13084851	4,115,819	1.3
4	1.68	rs7671597	41,665,756	rs2035906	45,815,310	4.1
5	1.68	rs12110158	78,378,722	rs257239	97,997,738	19.6
6	1.67	rs1322633	125,082,133	rs1490388	126,514,509	1.4
11	1.64	rs1320211	15,301,410	rs10833818	22,795,026	7.5
15	1.68	rs937302	33,627,848	rs11070349	41,792,819	8.2
20	1.41	rs237417	5,770,142	rs4140471	7,226,740	1.5

**Table 2:** Eight variants identified by whole exome sequencing

Chr	Gene	REF	ALT	SNP Position	dbSNP	ExAC MAF (%)	Protein Effect
6	*HEY2*	A	AC,C	126,080,841	None	None	INDEL
3	*WDR48*	GTTTTG	GTTT, GTTTG	39,136,139	None	None	INDEL
3	*RPL14*	A	ACTGCTG	40,503,520	None	None	INDEL
5	*MTX3*	G	C	79,281,458	None	None	Missense
5	*ADGRV1*	C	T	90,144,496	rs747622607	0.003235	Nonsense
5	*SPATA9*	A	G	95,011,189	rs55796768	0.958466	Missense
5	*ERAP1*	C	G	96,139,250	None	None	Missense
15	*GOLGA8A*	C	T	34,673,722	rs76522922	None	Missense

At the start of this study, we were aware of hearing loss (HL) in three sibships with varying audioprofiles, including two sisters who are the product of a consanguineous union. On the basis of the pending molecular diagnosis of Usher syndrome and the serious prognosis, the clinic contacted the sisters in order to request a visual examination (PID V-2 and V-3; Fig. 1a). The sisters are now in their late third and early fourth decade. Both women report impaired vision for some years. Ophthalmology reports on both sisters indicated definite features of RP. Further testing of PID V-3 identified bone spicule pigmentation of the retina (Fig. 2b) and a significant reduction in peripheral visual acuity (Fig. 2c), which are consistent with “typical RP”. These findings prompted the clinic to counsel the women regarding their new diagnosis of USH2C.

### *USH2A* c.5777-1G > A and c.10388-2A > G genotype/phenotype analyses

The comprehensive gene panel that was offered to the clinical case of the 3-year-old child diagnosed with isolated hearing loss at 3 months (Family 4110; Fig. 1c) identified two novel *USH2A* splicing variants: c.5777-1G > A (Fig. 2d) and c.10388-2A > G (Fig. 2e). Cascade sequencing confirmed the maternal contribution as c.5777-1G > A and the paternal contribution as c.10388-2A > G, and verified these novel variants reside *in trans*. However, given this child’s young age and the novelty (variants of unknown significance) of the *USH2A* variants, the genetic testing results are of limited value.

Fortuitously, the targeted USH gene panel offered to Family R0723 identified these same *USH2A* splicing variants. The proband (PID II-5) and his brother (PID II-3) are homozygous for *UHS2A* c.5777-1G > A, their nieces (PIDs III-1 and III-2) are compound heterozygotes (c.5777-1G > A; c.10388-2A > G; Fig. 1e). Even though the deceased brother (PID II-6) was not available for genetic testing, he is likely a *USH2A* c.5777-1G > A homozygote, given the strong family history of RP. Retrospective audiological data on the proband’s niece, PID III-1, from mid-third decade to mid-fifth decade show a stable hearing loss according to GenDeaf guidelines (Fig. 1d) [15]. The proband (PID II-5) has moderate to severe hearing loss in his seventh decade, not significantly worse than his younger niece (PID III-1) in her late fifth decade (Fig. 1d). His other niece, PID III-2, reveals a similar clinical phenotype (data not shown). With respect to RP, the proband (PID II-5) and his two brothers (PID II-3 and II-6) reported decreased night vision by their late 20s (Fig. 1e); however, RP as seen in retinal photographs of the proband was not diagnosed in the brothers until their late 40’s (Fig. 2f). Following the diagnosis of RP in the uncles, their nieces who had documented hearing loss were closely monitored, and reduced visual fields noted at age 14 in PID III-2, indicating the first symptoms of RP. Abnormal dark adaptation and ERG responses were recorded in both nieces in the third decade and retinal photographs of PID III-2 illustrate arterial attenuation, a characteristic sign of early RP (Fig. 2f).

### *USH2A* c.5777-1G > A and c.10388-2A > G experimental validation of splicing effects

**Fig. 3:**
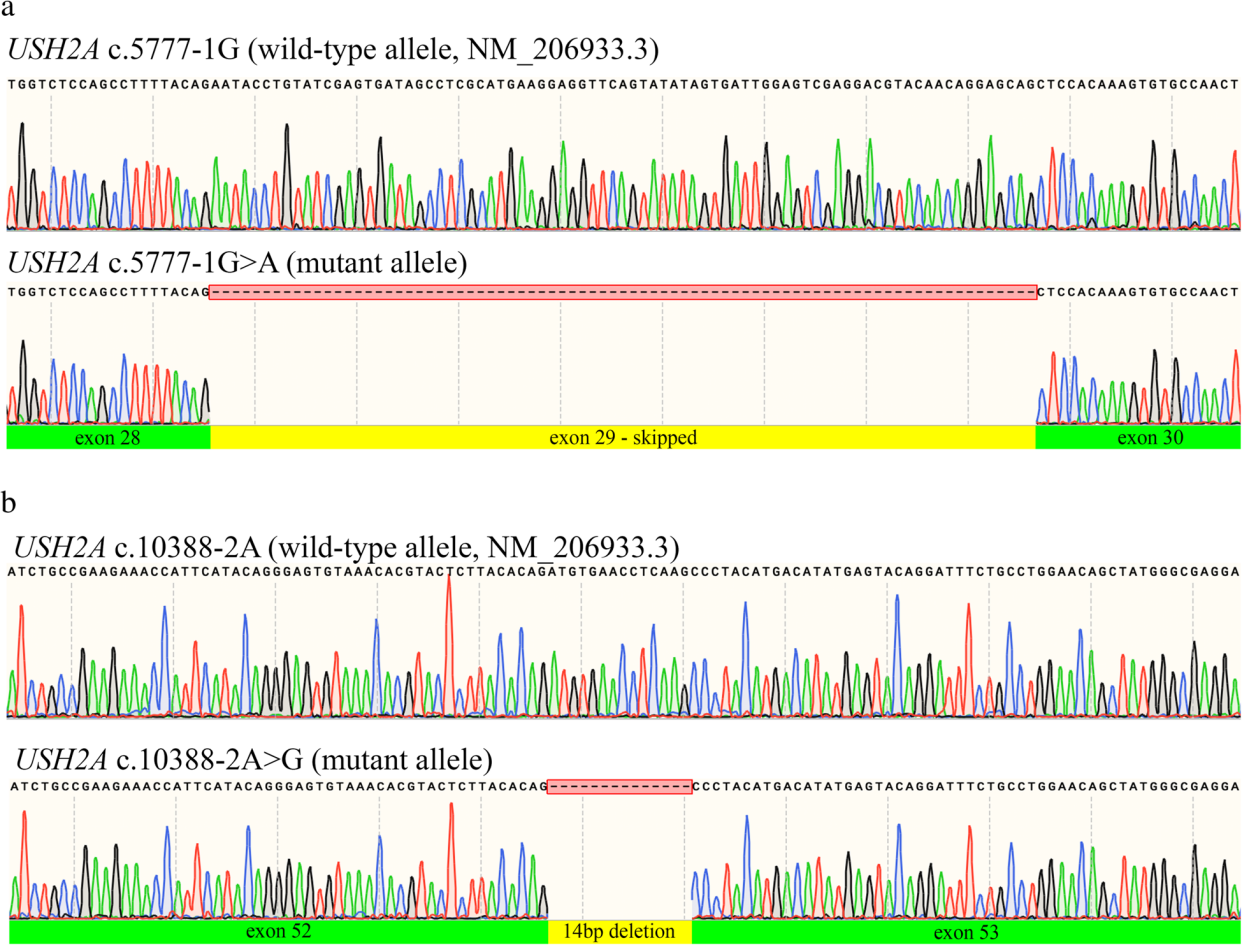
Sequence electropherograms of *USH2A* c. 5777-1G > A and 10,388-2A > G RT-PCR products. **a)** *USH2A* c. 5777-1G > A causes exon skipping and the in-frame deletion of 81 bp, **b)** *USH2A* 10,388-2A > G activates a cryptic acceptor splice site, resulting in the loss of 14 bp and a frameshift (p. Asp3463Alafs*6)

Cascade sequencing revealed that both *USH2A* c.5777-1G > A and *USH2A* c.10388-2A > G co-segregate with disease in families R0723 and R4110. In silico analyses using Alamut Visual suite of algorithms predicted that both variants cause exon skipping (MaxEnt: -100.0%, NNSPLICE: -100.0% and SSF: − 100.0%). Using patient-derived cells, Sanger sequencing of cDNA confirmed that *USH2A* c.5777-1G > A causes the skipping of exon 29 leading to an in-frame deletion (p. Glu1926_Ala1952del) in an affected individual (PID III-2) compared with a control sample (Fig. 2d; Fig. 3a). The sequencing of patient cDNA also determined that *USH2A* c.10388-2A > G activates a cryptic acceptor site 14 bps downstream of the canonical splice site (Fig. 2e; Fig. 3b), resulting in a premature stop codon (p.Asp3463Alafs*6). Based on cascade sequencing within these families and subsequent RNA analysis, *USH2A* c.5777-1G > A and c.10388-2A > G can both be classified as pathogenic variants according to the ACMG guidelines (PVS1, PS3, PM2, PM3, PP1, PP3) [16].

## Discussion

Two novel pathogenic variants in *USH2A* account for cases recruited or referred as isolated hearing or vision loss in two families in this study. Clinical evidence suggests that the two novel *USH2A* pathogenic variants result in congenital moderate to severe HL, and RP in the pre/post-pubertal period; findings similar to that of previously reported pathogenic variants in *USH2A* [17]. Several affected family members present as compound heterozygotes, suggesting that both *USH2A* c.5777-1G > A and c.10388-2A > G pathogenic variants are sufficient to cause USH2A and therefore are USH2A-specific. This finding is consistent with the allelic hierarchy model of *USH2A* alleles, which suggests that certain alleles are USH2A-specific and others RP-specific, and the presence of at least one RP-specific allele causes isolated RP with normal hearing [18]. Given our clinical case with a young girl who also tested positive for both of these alleles, we are now increasing surveillance for visual symptoms, leading to improved management of USH.

Similarly, we find that two sisters with hereditary hearing loss, the product of a second cousin union, are homozygous for a nonsense pathogenic variant in *ADGRV1* (c.17062C > T, p.Arg5688*). Visual examination secondary to molecular analyses confirmed typical RP (late third and fourth decade) in addition to hearing loss (first decade) and prompted a corrected USH diagnosis. This is consistent with previous reports of *ADGRV1* pathogenic variants associated with early onset of hearing loss with delayed visual impairment [19, 20], most of which are located in the calx-β motif [21], and the *ADGRV1* c.17062C > T lies downstream (3′) to this calx-β motif. This variant is rare, and to our knowledge, has only been reported once before when it was identified in 1/31 French non-*USH2A* patients [22]. In addition to causing USH2C, nonsense *ADGRV1* pathogenic variants have been shown to cause dominant audiogenic epilepsy [23, 24]. However, the two affected sisters from R2100 whom are homozygous for *ADGRV1* c.17062C > T do not present with audiogenic epilepsy.

Clinically, USH2 should be suspected in patients with bilateral, congenital, sensorineural, mild to severe hearing loss, normal vestibular function, and post-pubertal RP, most often in the second decade [4, 26]. Visual examinations revealed a ‘typical RP’ phenotype in patients diagnosed with USH2A or USH2C [21]. Likewise, from an audiological standpoint, our data is consistent with previous reports of a stable moderate to severe hearing loss [19, 22, 27, 28]. These results indicate that USH2A and USH2C are not readily discerned phenotypically [21]. The USH2A, ADGRV1 and WHRN proteins co-localize at the stereocilia base in developing cochlear hair cells and together form the Ankle-link complex at the base of sensory hair cells and at the periciliary membrane complex of photoreceptors [29–31], so it is not surprising that the USH2A and USH2C phenotypes are indistinguishable.

To determine variant pathogenicity, clinical best-practice guidelines, such as ACMG [16] and EuroGentest [25] are important to follow. For the splicing variants, we used in silico prediction algorithms for preliminary assessment only, and experimentally confirmed the splicing effects using patient-derived B-cell lines. For the nonsense variant, we confirmed that the parents were unaffected carriers and that their affected children received one copy of the novel nonsense *ADGRV1* c.17062C > T variant from each of them, establishing that we are detecting two disease alleles *in trans* and confirming the recessive pattern for Usher syndrome.

In a recent meta-analysis including all of the known genes causing usher syndrome, *USH2A* (50%) mutations are the most common with *ADGRV1* mutations being less frequent (5%) in patients with both visual and hearing impairments [32]. In patients with seemingly isolated sensorineural deafness, 7.5% had disease-causing mutations in USH genes, and are therefore at high risk of developing RP. In isolated cases of ‘hearing loss’ or ‘vision loss’, it is important to screen both USH and RP genes, as an accurate diagnosis of Usher syndrome is essential for patient clinical follow-up, particularly the referral and access to the correct support systems.

## Conclusions

Recognition of syndromic forms of both hearing and vision loss, especially Usher syndrome, is important given the major impact of these types of sensory losses on the acquisition of speech in children and quality of life for adults. In this report, USH was not considered in these cases until genetic testing was performed. Close collaboration between local clinics and molecular genetics researchers was necessary to fully categorize three novel USH variants as pathogenic using ACMG criteria. Accurate molecular diagnosis of patients is essential to provide new opportunities for patients and their families to enroll in therapeutic trials.

## Abbreviations

DNA: Deoxyribonucleic acid
LOD: Logarithm of odds score
NL: Newfoundland and Labrador
PID: Pedigree identification number
RP: Retinitis pigmentosa
RT-PCR: Reverse-transcription polymerase chain reaction
USH: Usher syndrome
USH2A: Usher syndrome type 2A
USH2C: Usher syndrome type 2C
